# Editorial: Ecosystem and planetary health and emerging/re-emerging zoonoses

**DOI:** 10.3389/fvets.2025.1748617

**Published:** 2025-12-16

**Authors:** Demelash Areda, Bedaso Edao

**Affiliations:** 1 School of Art and Science, Ottawa University, Surprise, AZ, United States; 2 College of Veterinary Medicine, University of Illinois at Urbana-Champaign, Urbana, IL, United States

**Keywords:** emerging zoonoses, planetary health, one health, ecosystem, environmental drivers

## Introduction

Planetary health is the practice of ensuring the highest possible levels of health, wellbeing, and equity for all humankind through proper management of political, economic, and social systems that influence human progress, and the Earth's natural systems that provide the environmental boundaries necessary for human life to thrive (1). In the past 100–150 years, humans have utilized the Earth's resources to enhance their quality of life and significantly extend their lifespan (2). However, this has reached a tipping point where environmental exploitation has begun to adversely affect human health (2). Understanding the connections between ecosystem integrity, planetary health, and emerging zoonoses is essential, as environmental disturbances directly increase the risk and consequences of infectious disease epidemics. Identifying these relationships enhances our capacity to avert and address forthcoming health concerns.

The Research Topic, *Ecosystem and Planetary Health and Emerging/Re-emerging Zoonoses*, comprises six studies that investigate the epidemiology and risk factors of various zoonotic pathogens, including avian influenza, *Bartonella* spp., *Leptospira* spp., invasive species, and zoonotic hazards to vulnerable human populations. The six contributions were grouped into the following three main themes based on their focus: (1) Pathogen transmission across wildlife, livestock, and human populations, along with the ecological factors that shape these interfaces; (2) the influence of environmental change, landscape disturbance, biodiversity loss, and climate variability on the emergence and spread of zoonotic pathogens; and (3) human vulnerability within the broader context of declining planetary health.

## Thematic summary of the published articles

### Zoonoses at the wildlife–livestock–human interface

The study by Islam et al. investigated the temporal patterns, prevalence, and risk factors of A/H5 and A/H9 subtypes within the turkey trading network in Dhaka, Bangladesh, employing a combination of molecular, spatial, and statistical tools. The findings from the article reveal the persistent transmission of avian influenza A/H5 and A/H9 subtypes throughout turkey trading networks in Dhaka's live bird markets, Bangladesh, attributed to the introduction of dead or sick birds into the area. These results emphasize the importance of improving disease control and preventive programs through consistent surveillance of livestock trading routes and networks, in addition to addressing biosecurity weaknesses that facilitate the transmission of infectious diseases.

Similarly, the study conducted by Viani et al. sought to identify the prevalent species of *Bartonella* spp. circulating in fox populations in the Piedmont and Aosta Valley regions of Italy. The study also explored environmental factors contributing to the survival and persistence of the pathogen in these areas, and it employed epidemiological and remote sensing tools to find a positive association between the presence of *Bartonella* species and environmental humidity, indicating that changing environmental conditions may play a pivotal role in shaping pathogen distribution and persistence. The authors also highlight that wildlife can serve as an important sentinel of ecosystem health and as an early warning indicator of changing environmental conditions.

### Environmental drivers and ecosystem disturbance

One of the three articles within this theme is research conducted by Min and Yoo, who investigated outbreaks of highly virulent avian influenza epidemics in South Korea. The investigators utilized epidemiological and statistical methods to examine the ecological factors contributing to avian influenza virus epidemics in the study area. The results show that reduced temperatures, increased farm density, and diminished predator species diversity were significantly associated with an elevated incidence of HPAI infection. The study predicted that horizontal transmission and spillover were the main pathways for HPAI dissemination in chicken and duck farms, respectively. These findings align with extensive ecological evidence indicating that biodiverse environments can have protective effects on infection spillover and transmission.

The second contribution to address this specific theme, authored by Mazzanti et al., sought to determine the prevalence and associated risk factors of Leptospira infection in beef cattle in Argentina. The study utilized a range of methodologies and tools, including serology, epidemiology, statistical modeling, GPS, and satellite technology. The study demonstrates the significant influence of climatic and hydrological variables on the prevalence of Leptospira infection. This aligns with the observation that alterations in global precipitation patterns, along with the heightened frequency and intensity of flooding primarily due to climatic variability, facilitate the geospatial spread and subsequent transmission of diseases, including Leptospira infection.

The third article, by Jimenez et al., discussed the impact of natural, anthropogenic, and climatic factors on human, animal, and environmental health within the unique ecosystem of the Galapagos Islands, which is endowed with abundant terrestrial and marine biodiversity. The Galapagos Islands also serve as a model ecosystem to demonstrate how anthropogenic activities may rapidly and adversely alter a fragile ecosystem, creating an area with heightened risks for both animals and humans. The study also suggests methods to address these challenges through integrated One Health approaches.

### Human vulnerability in the planetary health context

The study authored by Garcia-Sanchez et al. aimed to identify potential zoonotic disease pathogens and their infection prevalence among immunocompromised Spanish children and their cohabiting pets. The study also identified risks associated with pathogen colonization and infection among the studied groups. The study indicates that immunocompromised Spanish children and their pets harbored one or more potential pathogens (44.6% and 31.5%, respectively). However, shared pathogens were primarily limited to Blastocystis, with no confirmed zoonotic transmission identified between the two groups. The primary species identified in pets were *Staphylococcus pseudintermedius, Cryptosporidium, Enterocytozoon bieneusi*, and hepatitis E virus. *Clostridium difficile* was predominantly identified as a potential pathogen in the immunocompromised Spanish children included in the investigation. Despite substandard animal health practices or past exposure to zoonotic pathogens, the study indicates that no particular risk factors, including hygiene, diet, or pet care, were linked to a higher prevalence of reported pathogens in either group.

The authors concluded that having a family pet does not increase the risk of exposure to or infection by potential pathogens in immunocompromised Spanish children, provided that fundamental disease prevention and control methods are implemented.

## Synthesis and future directions

Collectively, these investigations demonstrate that the origin and transmission of zoonotic illnesses are not attributable to a single factor but rather to interactions among ecological, socio-demographic, climatic, and environmental factors. The risk of zoonotic disease emergence and transmission is exacerbated by several factors, including: (1) climate variability that expands the geographic ranges of pathogens, facilitating contact between novel hosts and pathogens, thereby promoting cross-species transmission (3); (2) heightened spillover of potential pathogens due to declining biodiversity (4); (3) intensified circulation and transmission of pathogens resulting from increased global mobility and trade. It is therefore recommended that climate-informed One Health programs be implemented to address these timely challenges.
